# Treatment of Huge Hepatocellular Carcinoma Using Cinobufacini Injection in Transarterial Chemoembolization: A Retrospective Study

**DOI:** 10.1155/2016/2754542

**Published:** 2016-05-17

**Authors:** Jun Dong, Xiaofeng Zhai, Zhe Chen, Qun Liu, Hua Ye, Wei Chen, Changquan Ling

**Affiliations:** ^1^Shanghai University of Traditional Chinese Medicine, Shanghai 201203, China; ^2^Department of Integrative Oncology, Changhai Hospital of Traditional Chinese Medicine, Second Military Medical University, Shanghai 200433, China; ^3^Department of Radiology, Changhai Hospital, Second Military Medical University, Shanghai 200433, China

## Abstract

The aim of this study is to examine the safety and efficacy of Cinobufacini injection in transarterial chemoembolization (TACE) for treatment of huge hepatocellular carcinoma (HCC). Clinical data of 56 consecutive patients with HCC larger than 10 cm who had been treated with TACE between December 2010 and August 2014 were retrospectively analyzed. Among these patients, 31 belonged to the Cinobufacini group and 25 belonged to the epirubicin group. The clinical efficacy, survival time, and adverse events in patients in the two groups were compared. The objective response rate in the Cinobufacini group was significantly higher than that in the epirubicin group (53.6% versus 23.1%, *P* = 0.022). The median survival time (10.6 versus 14.1 months, *χ* ^2^ = 0.092, *P* = 0.762) and the median time to progression (4.9 versus 5.7 months, *χ* ^2^ = 0.097, *P* = 0.756) were similar between the groups. The incidence rate of adverse events was lower in the Cinobufacini group than in the epirubicin group (*P* < 0.05). The short-term clinical efficacy of Cinobufacini is better than that of epirubicin in TACE for treating huge HCC, while their long-term clinical efficacy is similar. However, lower incidence of adverse events was noted in TACE using Cinobufacini rather than epirubicin.

## 1. Introduction

Patients with a tumor larger than 10 cm, which is referred to as huge hepatocellular carcinoma (HCC), are generally at the advanced or late stage of the disease, with poor prognosis [1]. Their survival time is considerably shorter than that of patients with relatively small HCC [2]. In fact, huge HCC is very difficult to treat and no standard treatment strategy is available [3].

Transarterial chemoembolization (TACE) is an important palliative treatment method for patients with unresectable HCC. All patients with Barcelona Clinic Liver Cancer (BCLC) stages' A–C HCC are candidates for TACE treatment in Asia and most parts of North America [4]. The local clinical efficacy of TACE in obvious shrinking of tumor has been demonstrated. However, the long-term efficacy of TACE has not yet been investigated in detail. Previous studies showed that the 5-year survival rate of patients with huge HCC who underwent TACE treatment was lower than 10% [5, 6]. Besides, choosing the best chemotherapeutic drugs for this procedure is still controversial. Cisplatinum, epirubicin, and fluorouracil are the most commonly used chemotherapeutic drugs in TACE; however, the most effective drug among these to treat huge HCC is yet to be determined [7].

Traditional Chinese medicine (TCM) is an important approach in tumor treatment in China. Cinobufacini injection (Cinobufacini), an antitumor TCM preparation, is a water soluble extract of* Bufo gargarizans* that is especially suitable for patients with HCC [8]. Clinical studies have shown that application of Cinobufacini could increase the efficacy of TACE, reduce adverse events, and improve the immunity of the patients [9]. In China, Cinobufacini was mainly administrated intravenously during TACE; however, it is still unclear whether the efficacy of TACE and associated adverse events could be affected when Cinobufacini is used instead of chemotherapeutic drugs in TACE. In the present study, the clinical efficacy and adverse events of Cinobufacini and chemotherapeutic drugs in TACE for treatment of huge HCC were retrospectively analyzed.

## 2. Materials and Methods

### 2.1. Study Design

Data from 56 consecutive patients with huge HCC who were treated with TACE in the Department of Traditional Chinese Medicine, Changhai Hospital, Shanghai, between December 2010 and August 2014 were retrospectively analyzed. Among these patients, 31 belonged to the Cinobufacini group (27 male and 4 female; mean age 54.1 ± 11.2 years) and 25 belonged to the epirubicin group (all male; mean age 58.1 ± 12.4 years).

### 2.2. Diagnostic Criteria

HCC was diagnosed according to the diagnostic criteria detailed in the Expert Consensus of the Standard Diagnosis of Primary HCC issued in 2009 [10].

### 2.3. Inclusion and Exclusion Criteria

Patients aged between 18 and 75 years, those with unresectable HCC but with indications for TACE, those in whom the tumor diameter was ≥10 cm, those with BCLC stage B or C HCC and in whom the portal vein was not completely obstructed, those with hepatic functional reserve of Child-Pugh class A or B, and those with Eastern Cooperative Oncology Group (ECOG) score <3 were included in the study. Meanwhile, patients who were participating in any other drug trial, those who had a history of malignant tumor within the 5 years preceding the study, pregnant or breastfeeding women, and patients prone to allergies were excluded from the study.

The study was approved by the Hospital Ethics Committee and all patients signed informed consents.

### 2.4. Procedures

The femoral artery was punctured unilaterally and the Seldinger technique was used to pass the Cobra catheter over a guide wire. Common hepatic artery angiography was then performed to detect the exact location of the liver tumor, tumor-supplying blood vessels, portal vein thrombosis, and the existence of arteriovenous fistula. The Cobra catheter was further delivered to the proper hepatic artery or right/left hepatic artery and the mixture of Cinobufacini or epirubicin and Lipiodol was injected. All blood vessels in the tumor were superselected, and the procedures were performed by interventional experts from the Changhai Hospital under digital subtraction angiography (DSA). The time between two TACE procedures was 1.5–3 months.

### 2.5. Drug Combinations

In both patient groups, ondansetron hydrochloride was used to prevent vomiting, pantoprazole was used to inhibit gastric acid secretion, and reduced glutathione was used for liver protection. Other supportive treatments were also used.

### 2.6. Follow-Up

All patients were followed up after the TACE treatment till death or till the censor time of the study (December 2014). Data regarding the disease status and survival of the patients were collected every 3 months.

*Objective Response Rate of the Tumor*. Enhanced Computer Tomography (CT) or Magnetic Resonance Imaging (MRI) scanning of the patients was performed at 6–8 weeks after the TACE, and the clinical efficacies were evaluated according to the Modified Response Evaluation Criteria in Solid Tumors (mRECIST) [11]. Complete response (CR) indicates the disappearance of any intratumoral arterial enhancement in all target lesions; partial response (PR) indicates that the sum of the diameters of the target lesions (enhanced images in the arterial phase) decreased by ≥30%; stable disease (SD) indicates that the change of the target lesions do not qualify for either partial response or progressive disease; and progressive disease (PD) indicates that the sum of the largest diameters of the target lesions increased by at least 20% as compared with the baseline sum of the largest diameters of the tumor or the appearance of new lesion. Objective response rate (OR) indicates the percentage of the CR + PR in all the patients, and disease control rate (DC) indicates the percentage of the OR + SD in all the patients.

*Overall Survival and Time to Progression*. The overall survival (OS) referred to the time from the treatment to the death of the patient (caused by any cause). The time to progression (TTP) referred to the time from the treatment to the objective progression of the tumor.

*Grading of the Adverse Events*. The liver function, hematologic system, and clinical symptom-related adverse events were assessed on the day 3 after TACE according to the Standards of the Common Terminologies for Adverse Events (3rd edition) issued by the National Cancer Institute.

### 2.7. Statistical Analysis

SPSS version 21.0 was used for statistical analysis. The Kaplan-Meier method was used to calculate survival curves, and the log-rank test was used for survival comparisons. Quantitative data in normal distribution were represented as means and standard divisions and compared using *t*-test, while quantitative data in nonnormal distribution were compared using the Wilcoxon rank-sum test. Qualitative data were compared using Pearson's chi-square test or Fisher's exact test. *P* < 0.05 was considered statistically significant.

## 3. Results

### 3.1. Patient Characteristics

From December 2010 to August 2014, 56 consecutive patients with huge HCC who were treated with TACE in the Department of Traditional Chinese Medicine, Changhai Hospital, Shanghai, were retrospectively analyzed. Among these patients, 31 belonged to the Cinobufacini group (27 male and 4 female; mean age 54.1 ± 11.2 years) and 25 belonged to the epirubicin group (all male; mean age 58.1 ± 12.4 years).

The characteristics of patients in two groups in terms of age, sex, tumor size, alpha-fetal protein (AFP) levels, total bilirubin (TB), alanine transaminase (ALT) levels, and the BCLC stage, ECOG score, and the Child-Pugh classification were summarized in Table 1.

### 3.2. Clinical Efficacy

CT and MR imaging results showed that the objective response rate in the Cinobufacini group was significantly higher than that in the epirubicin group (*P* = 0.028); however, the disease control rate was not significantly different between the groups (*P* = 0.162) (Table 2). Besides, the AFP level was 567 ± 749 and 571 ± 726 in the Cinobufacini and epirubicin groups, respectively, and the difference was not statistically significant (*P* = 0.654) (Table 3).

### 3.3. Overall Survival and Time to Progression

**Figure 1:**
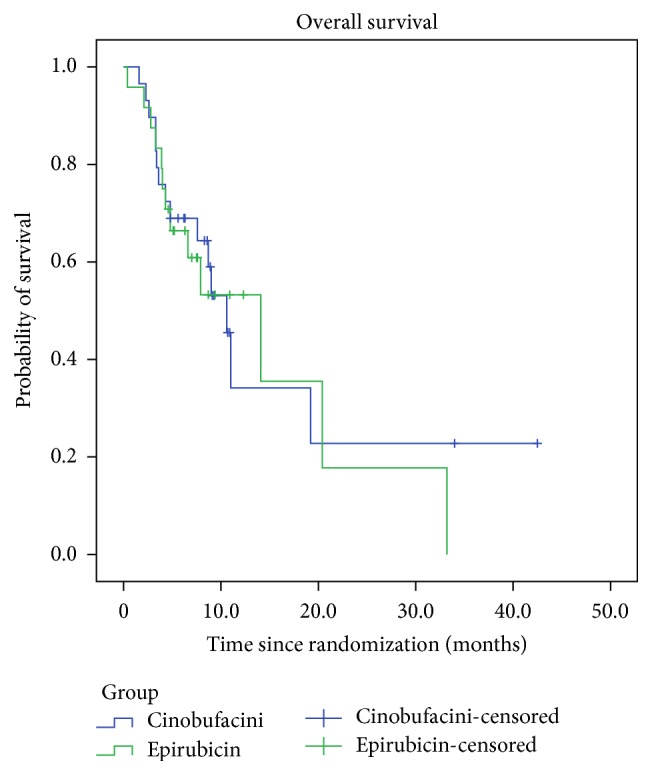
Kaplan-Meier estimates of overall survival of patients with HCC.

**Figure 2:**
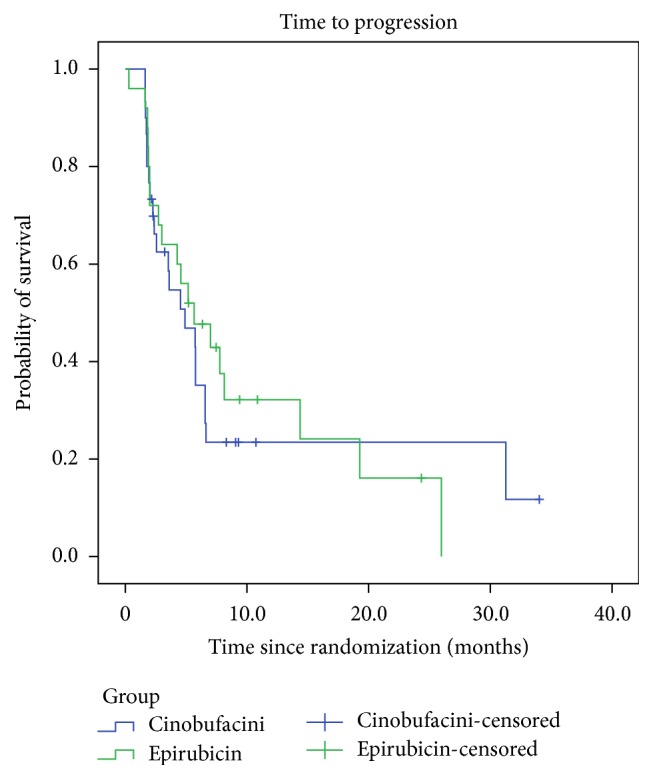
Kaplan-Meier estimates of time to progression of HCC.

The median OS period was 10.6 (95% CI: 8.5–12.6) months and 14.1 (95% CI: 4.6–23.6) months in the Cinobufacini and epirubicin group, respectively, and the difference between the groups was not statistically significant (*χ* ^2^ = 0.092, *P* = 0.762) (Figure 1). The median TTP was 4.9 (95% CI: 2.3–7.5) months and 5.7 (95% CI: 2.0–9.3) months in the Cinobufacini and epirubicin groups, respectively, and the difference was not statistically significant (*χ* ^2^ = 0.097, *P* = 0.756) (Figure 2).

The 1- and 2-year survival rates were 69% and 45.5% in the Cinobufacini group and 66.4% and 17.8% in the epirubicin group. No significant difference was found in the 1- and 2-year survival rates between the two groups (*P* = 0.708 and 0.058, resp.).

### 3.4. Adverse Events

#### 3.4.1. Liver Function

The frequency and degree of increase in TB and ALT levels on day 3 after TACE were significantly lower in the Cinobufacini group than in the epirubicin group (*P* < 0.05), while the frequency and degree of increase in aspartate aminotransferase (AST) level were not significantly different between the groups (*P* > 0.05) (Table 4).

#### 3.4.2. Hematologic System

The frequency and degree of decrease in white blood cells (WBC) and platelets (PLT) count on day 3 after TACE were significantly lower in the Cinobufacini group than in the epirubicin group (*P* < 0.05), while the frequency and degree of decrease in hemoglobin (HGB) were not significantly different between the groups (*P* > 0.05) (Table 4).

#### 3.4.3. Clinical Symptoms

The incidence of liver pain and abdominal distension in the epirubicin group was significantly higher than that in the Cinobufacini group (*P* < 0.05). No nausea or vomiting was observed in the Cinobufacini group, while its incidence in the epirubicin group was 36.0%, which was significantly higher than that in the Cinobufacini group (*P* < 0.05). Fever was noted in both groups, and the incidence was not significantly different between the groups (*P* > 0.05) (Table 4).

## 4. Discussion

Because of vascular infiltration and formation of satellite lesions, patients with huge HCC are generally at the advanced or late stage of the disease. According to BCLC stage system, these patients belong to C stage, who are not recommended to receive TACE treatment in most guidelines issued by American Association for the Study of Liver Diseases (AASLD) and European Association for the Study of the Liver (EASL) and others [12, 13]. One of the main reasons is that patients with huge HCC always have poor liver function, while the chemotherapeutic drugs used in TACE may further aggravate the liver damage. Some other studies also showed that HCC was resistant to multiple chemotherapeutic drugs, while the efficacy of TACE and transarterial embolization (TAE), which is mainly dependent on Lipiodol embolization, in treating liver cancer was not significantly different. Even randomized clinical trials failed to demonstrate the superiority of TACE over TAE [14]. Therefore, in this study, we designed that using Cinobufacini with similar antitumor effects but less adverse effects instead of chemotherapeutic drugs in TACE for treating patients with huge HCC. The findings of the present study showed that the objective response rate of using Cinobufacini in TACE was 53.6%, and the median patient survival time was 10.6 months; this suggested that using Cinobufacini in TACE could improve its clinical efficacy, resulting in a long-term survival similar to that observed using interventional therapies with western medicines. As most of the patients with huge HCC have poor liver function, perfusion of chemotherapeutic drugs may not be beneficial enough; this result revealed that using Chinese herbs and other antitumor drugs as complementary and alternative therapies may get a better future.

Cinobufacini injection used in our study is extracted from traditional Chinese medicine* Bufo gargarizans* and is approved by the Chinese Food and Drug Administration since 1991 for antivirus and antitumor treatments. It was reported that Bufalin, resibufogenin, and cinobufagin are the three major materials to which the anticancer activity of Cinobufacini can be attributed [15].* In vitro* studies demonstrated that Cinobufacini has evident antitumor effects, which could inhibit the syntheses of tumor cell DNA and RNA and thus affect the expression of oncogenes [16]. Some studies revealed that the growth of human hepatocellular cell lines (MGC-80-3 and SMMC-7721) could be inhibited by Cinobufacini and that this was mediated through S-phase arrest and inhibition of bcl-2 expression [17]. Recent studies demonstrated that Cinobufacini inhibits the viability of HepG2 cells via cytoskeletal destruction and cell membrane toxicity [18]. Some researchers also investigated possible apoptotic mechanisms induced by Cinobufacini in HCC cell lines HepG(2) and Bel-7402 and the results suggested that Cinobufacini could induce apoptosis of HCC cells through mitochondria- and Fas-mediated caspase-dependent pathways with the increase of treatment time [19]. Clinical studies have shown the antitumor effects of Cinobufacini against liver and lung cancers [20]. The combined use of Cinobufacini with chemotherapy and/or radiotherapy could reduce the toxic effects and increase patient immunity [21]. In addition, Cinobufacini injection has been proved to diminished risk of recurrence of small-sized HCC after resection [22]. These results may explain the antitumor effect of Cinobufacini observed in our study.

Meanwhile, Cinobufacini could protect liver function and improve quality of life [23]. The characteristics of Chinses HCC patients are often combined with different degree of hepatitis and liver cirrhosis [24], as well as abnormal liver function. This condition is more obvious in huge HCC patients at advanced stage. ALT and other indexes elevated in most patients observed in our study. In China, Cinobufacini was mainly administrated intravenously during TACE. Clinical study have shown that Chinese medicine taken in combination with TACE has beneficial effects on reduction of toxicity compared with TACE alone [25]. Introduction of Cinobufacini via the hepatic artery not only is an effective local and systemic treatment but also has antitumor and liver-protective effects, consequently increasing the clinical efficacy of TACE and decreasing the adverse effects.

In the present study, no severe adverse effect was observed in patients who underwent TACE with Cinobufacini, and the effects on the liver function and hematologic system and clinical symptoms were significantly lower than those observed when epirubicin was used, suggesting that Cinobufacini has obvious benefits in reducing TACE-related adverse effects. Huge HCC could continuously aggravate liver cirrhosis and portal hypertension; in addition, it does not respond to chemotherapeutic drugs. Therefore, repeated TACE could result in significant drug resistance and liver function impairment; massive hemorrhage of gastrointestinal tract and liver failure could also occur in severe cases. Meanwhile Cinobufacini may synergistically enhance the efficacy of TACE and reduce its toxicity.

Another advantage of using Cinobufacini instead of chemotherapeutic drugs observed in our study is that Cinobufacini has strong blood vessel contraction effect. The key point of TACE treatment is effective embolism of the blood supplying the major artery and collateral arteries. However, the blood supply in huge HCC is very complex; several collateral arteries could participate in the blood supply to the tumor, and additional collateral circulation could be formed after the embolism, while the collateral arteries are generally too small to be embolized. In addition, the existence of undetectable hematogenous spread could result in cancer metastases to other organs shortly after the operations. In this study we also observed that Cinobufacini could effectively contract the blood vessels through DSA in the treatment procedures; thus, the diffusion of Cinobufacini in the tumor-supplying blood vessels after injection through the hepatic artery may also potentially contract the small blood vessels, resulting in better embolism effects.

## 5. Conclusions

In summary, the findings of the present study showed that treating huge HCC with Cinobufacini injection in TACE is safe and effective and could be a useful antitumor treatment combining traditional Chinese medicine and western medicine. However, the present study was limited by the retrospective design, relatively small sample size, and observations restricted to huge HCC. More randomized controlled trials with larger sample sizes and those including patients with other types of primary HCC are required in order to provide more reliable evidence.

## Figures and Tables

**Table 1:** Comparison of baseline data between the groups.

	Cinobufacini group (*n* = 31)	Epirubicin group (*n* = 25)	*P* value
Age	54.1 ± 11.2	58.1 ± 12.4	0.583
Sex			0.120
M	27 (87.1)	25 (100)	
F	4 (12.9)	0 (0)	
BCLC stage			0.611
B	17 (54.8)	12 (48.0)	
C	14 (45.2)	13 (52.0)	
Child-Pugh stage			0.443
A	26 (83.9)	23 (92.0)	
B	5 (16.1)	2 (8.0)	
ECOG score			0.981
0	13 (41.9)	10 (40.0)	
1	17 (54.8)	14 (56.0)	
2	1 (3.2)	1 (4.0)	
AFP			0.386
<400	15 (48.4)	15 (60.0)	
≥400	16 (51.6)	10 (40.0)	
HBsAg			0.153
+	28 (90.3)	18 (72.0)	
−	3 (9.7)	7 (28.0)	
TB	16.9 ± 7.8	16.3 ± 7.6	0.653
ALT	46.3 ± 29.2	51.2 ± 44.0	0.351
Tumor size	12.1 ± 3.2	11.8 ± 4.0	0.760

**Table 2:** Comparison of clinical efficacy of TACE between the groups.

Group	Number of patients (%)				
	PR	SD	PD	OR	DC
Cinobufacini group (*n* = 31)	15 (48.4)	10 (32.3)	6 (19.4)	15 (48.4)^*∗*^	25 (80.6)
Epirubicin group (*n* = 25)	5 (20.0)	11 (44.0)	9 (36.0)	5 (20.0)	16 (64.0)

^*∗*^ *P* < 0.05, comparing with the epirubicin group.

PR: partial response; SD: stable disease; PD: progressive disease; OR: objective response; DC: disease control.

**Table 3:** Comparison of the AFP level between the groups.

	Cinobufacini group (*n* = 31)	Epirubicin group (*n* = 25)	*P* value
Before treatment	885 ± 931	596 ± 690	0.256
After treatment	567 ± 749	571 ± 726	0.654

**Table 4:** Comparison of the incidence of adverse events between the groups.

Parameter	Cinobufacini group (*n* = 31)				Epirubicin group (*n* = 25)			
	Total	Grade 1	Grade 2	Grade 3	Total	Grade 1	Grade 2	Grade 3
Liver function								
TB	18 (58.0)	13 (41.9)	5 (16.1)	0 (0.0)	21 (84.0)	4 (16.0)	15 (60.0)	2 (8.0)
ALT	16 (51.7)	8 (25.8)	6 (19.4)	2 (6.5)	22 (88.0)	6 (24.0)	11 (44.0)	5 (20.0)
AST	18 (58.1)	9 (29.0)	6 (19.4)	3 (9.7)	20 (80.0)	11 (44.0)	6 (24.0)	3 (12.0)
Hematologic system								
WBC	1 (3.2)	1 (3.2)	0 (0.0)	0	8 (32.0)	6 (24.0)	2 (8.0)	0
HGB	6 (19.3)	5 (16.1)	1 (3.2)	0	5 (20.0)	5 (20.0)	0	0
PLT	5 (16.1)	4 (12.9)	1 (3.2)	0	14 (56.0)	9 (36.0)	2 (8.0)	3 (12.0)
Clinical symptom								
Fever	14 (45.2)	—	—	—	17 (68.0)	—	—	—
Liver pain	7 (22.6)	—	—	—	20 (80.0)	—	—	—
Abdominal distension	5 (16.1)	—	—	—	13 (52.0)	—	—	—
Nausea and vomiting	0 (0.0)	—	—	—	9 (36.0)	—	—	—

TB: total bilirubin; ALT: alanine aminotransferase; AST: aspartate aminotransferase; WBC: white blood cells; HGB: hemoglobin; PLT: platelets.
